# The effect of low-temperature-induced immune activation in elderly idiopathic facial palsy: the association between Th1/Th2 imbalance and facial nerve inflammation

**DOI:** 10.3389/fimmu.2026.1718030

**Published:** 2026-09-30

**Authors:** Xing Tang, Ziqin Ji, Xueling Xiao, Tongtong Zhu, Shoujen Lan, Yeayin Yen, Xin Shao, Shune Tan, Chao Wang, Jicheng Zhang

**Affiliations:** 1 Department of Traditional Chinese Medicine Rehabilitation, Chongqing Emergency Medical Center, Chongqing, China; 2 Chongqing Key Laboratory of Emergency Medicine, Chongqing University Central Hospital, Chongqing, China; 3 Medical College, Jiangsu University, Zhenjiang, Jiangsu, China; 4 Sub-health Clinical Medicine Research Center, Sichuan Integrative Medicine Hospital, Chengdu, China; 5 College of Acupuncture-Moxibustion and Tuina, Chengdu University of Traditional Chinese Medicine, Chengdu, China; 6 Department of Healthcare Administration, Asia University, Taiwan, China; 7 Department of Acupuncture, Sichuan Integrative Medicine Hospital, Chengdu, China

**Keywords:** elderly population, facial nerve inflammation, idiopathic facial palsy, immune aging, low-temperature exposure, Th1/Th2balance

## Abstract

The pathogenesis of idiopathic facial palsy in the elderly has not been fully elucidated. Recent studies have shown that exposure to low temperatures may participate in the pathological process through immune-mediated pathways. This article reviews that cold stimulation can disrupt the Th1/Th2 immune balance, manifested as reduced secretion of Th1-type cytokines and upregulation of Th2-type cytokines, thereby inducing peripheral inflammatory responses around the facial nerve, leading to nerve edema, demyelination, and conduction dysfunction. Due to the immune aging characteristics of the elderly population, including the reduction of the initial T cell pool, the imbalance of pro-inflammatory and anti-inflammatory factor ratios, and the decline in regulatory T cell function, they are more sensitive to the immune dysregulation induced by low temperatures and have a significantly increased risk of facial palsy. Clinical and animal experimental evidence supports this mechanism, suggesting that the serum IL-4/IFN-γ ratio may serve as a prognostic marker. Future multi-center studies are needed to verify the effectiveness of targeted immune regulation intervention through the Th2 pathway and explore the application value of low-temperature warning and individualized warming strategies in the prevention of facial palsy in the elderly.

## 1 Introduction

Idiopathic Facial Palsy (IFP) is a neurological disorder characterized by sudden paralysis of a single side of the facial muscles. Its incidence increases significantly with age (1). Epidemiological data show that the annual incidence rate among people over 60 years old can reach 45 per 100,000, which is much higher than the 15–20 per 100,000 among younger populations. Moreover, the incidence of IFP in the winter is 30% higher than that in the summer, suggesting that environmental factors may be involved in its onset. In the Kureiaat region of Saudi Arabia, the incidence of Bell’s palsy is approximately 25.7 cases per 100,000 people per year (2). A prospective study of 48 patients with Sudanese Bell’s palsy found a strong association between exposure to cold air and the occurrence of Bell’s palsy. In this study, the proportion of patients with a history of catching a cold before the onset of the disease reached 27.1% (3). It can be seen that exposure to cold air and the common cold are significant risk factors for Bell’s palsy.

However, traditional etiological studies have mostly focused on viral infections or genetic susceptibility, but these factors have limited explanatory power for elderly patients and cannot fully clarify their seasonal clustering and age-dependent characteristics. In recent years, the potential role of low-temperature exposure has gradually attracted attention (4). Clinical observations have found that the cold environment in winter is closely related to the acute onset of facial paralysis. Additionally, a cross-case study conducted in multiple hospitals analyzed the association between environmental temperature, atmospheric pressure, relative air humidity, or their 24-hour variations and the risk of hypertension in 825 patients with Bell’s facial paralysis or different subgroups. The impact of weather changes on risk varies among different populations with underlying diseases (5). The cross-case study in multiple centers found that in the subgroup of diabetic patients, rapid fluctuations in environmental temperature (whether a drop of >2.25 °C or an increase of 0.75-2.25 °C) would increase the risk of Bell’s facial paralysis by more than twice, while no such significant association was observed in the non-diabetic subgroup. This supports the idea that “diabetic facial paralysis” may represent a unique disease entity, where the neural response to temperature changes or metabolic adaptation capacity may be impaired, thereby amplifying the harm of cold stimulation. This finding suggests that when assessing the risk of cold exposure, the individual health status of the patient must be taken into account.

Animal experiments have further confirmed that low-temperature stimulation can induce a decrease in the conduction velocity of the facial nerve in mice and infiltration of inflammatory cells around blood vessels, simulating the pathological manifestations of human facial paralysis (6). However, how low temperature affects the facial nerve through the immune system still lacks a systematic integrated analysis.

Immune aging is a typical characteristic of the elderly population, manifested by a reduction in the initial T cell pool, excessive activation of memory T cells, imbalance in the ratio of pro-inflammatory to anti-inflammatory factors, and functional decline of Th cell subgroups (7). Cold, as a physical stressor, may further disrupt the already fragile immune balance of the elderly through activating cold receptors, regulating the sympathetic nervous system and the cytokine network, leading to excessive inflammatory responses around the facial nerve. Studies have shown that the Th2 immune response in aged mice undergoes significant changes, including imbalance in cytokine secretion, abnormal T cell polarization, and decreased immune memory function (8). These changes not only affect the defense ability of the elderly individuals against pathogens but also may lead to chronic inflammation or immunosuppression. Immune intervention for the elderly population should fully consider the age-related Th2 function remodeling, such as restoring Th2 effector function by targeting GATA3 chromatin remodeling or inhibiting the IFN-γ pathway (9). Understanding the regulatory mechanism of Th2 immune response in aged mice is of great significance for developing immune intervention strategies for the elderly population (10, 11).

However, most existing studies are limited to a single aspect, such as the effect of low temperature on blood vessels or the general changes in immune cells, and lack a comprehensive mechanism explanation from the entire chain of “low-temperature stimulation-immune imbalance -nerve damage”, especially specific analyses for the elderly population. Based on this, this review systematically summarizes the process of Th1/Th2 immune imbalance in elderly idiopathic facial nerve palsy induced by cold, and proposes the clinical application value of immune regulation and individualized prevention strategies, with the aim of providing new ideas for clinical intervention.

## 2 Immune activation effects of low-temperature exposure

### 2.1 Direct effects of low temperature: neurovascular-tissue damage cascade

The low-temperature environment triggers peripheral nerve input signals by activating the temperature-sensitive transient receptor potential melastatin 8 (TRPM8) channel and the transient receptor potential ankyrin 1 (TRPA1) channel. TRPM8 and TRPA1, as two different cold sensation receptors, play different but complementary roles in cold perception. TRPM8 mainly transmits non-painful cold sensations and its activation regulates the body’s physiological responses through the neuroendocrine pathway to cope with the low-temperature environment. While TRPA1 is more associated with cold-induced pain sensations, its activation may exacerbate the discomfort caused by the low temperature. Together, they constitute the complex perception and response system of the body to the low-temperature environment (12). This process causes facial blood vessel constriction, especially in the intracranial segment of the facial nerve, causing local microcirculation disorders. Animal experiments show that low-temperature exposure can reduce facial nerve blood perfusion by 40%, leading to ischemia of the nerve end membrane and accumulation of lactic acid, directly damaging the integrity of Schwann cell membranes, providing a pathological basis for subsequent inflammatory infiltration. Additionally, low temperature can induce the expression of apoptosis-related proteins in neurons through mitochondrial dysfunction, exacerbating nerve damage (13).

### 2.2 Indirect immune regulation by low temperature: dual inhibitory and pro-inflammatory effects

The regulation of the immune system by low temperature is bidirectional. On one hand, low temperature (4°C) can inhibit the expression of co-stimulatory molecules (CD80/CD86) on the surface of dendritic cells, reducing their antigen-presenting ability, and blocking the activation of naive T cells; at the same time, it promotes the expansion of myeloid inhibitory cells, inhibiting the proliferation of effector T cells by secreting IL-10 and TGF-β (5). On the other hand, low temperature can activate the NLRP3 inflammasome in innate immunity, promoting the release of IL-1β and IL-18 by macrophages, exacerbating local inflammatory responses. This contradictory effect is more pronounced in the elderly population. Their immune system has both dendritic cells function decline and excessive activation of the inflammasome, resulting in elevated basal pro-inflammatory factors (IL-6, TNF-α) levels, forming a special state of “inhibitory immunity” and “chronic low-level inflammation” (14).

### 2.3 Characteristics of immune response in the elderly population: immune aging amplifies the effect of low temperature

The core characteristics of immune aging in the elderly include a significant reduction in the initial T cell pool due to thymus atrophy, an increase in the proportion of memory T cells and their functional exhaustion, and a decline in the inhibitory ability of regulatory T cells (Treg) (15). Under low temperature exposure, elderly individuals are unable to effectively initiate an adaptive immune response due to the reduction in initial T cells. The excessive activation of memory T cells (especially Th2 type) leads to increased secretion of IL-4 and IL-13, further disrupting the Th1/Th2 balance (16). At the same time, aged macrophages have a lower response threshold to low temperature stimulation, and the NLRP3 inflammasome remains continuously activated, resulting in a 2-3-fold increase in the basal levels of pro-inflammatory factors (IL-6, TNF-α) compared to younger individuals, forming a vicious cycle of “low temperature - immune imbalance - neural inflammation” (**Table 1**). Clinical data show that the serum IL-4/IFN-γ ratio in elderly patients with facial paralysis is 1.8 times higher than that of healthy elderly individuals, suggesting that Th2 polarization is the key mechanism of low temperature-induced facial paralysis (17).

**Table 1:** Immune activation effects and neurological injury mechanisms caused by low-temperature exposure.

Mechanism classification	Specific process:	Key molecules/pathways	Effects on the nervous system
Direct effect: Nerve-vessel injury	Low temperature activates TRPM8 channel → sympathetic nerve excitation → release of catecholamines → contraction of facial blood vessels → local microcirculation disorder	TRPM8, norepinephrine, endothelin	The blood supply to the facial nerve is reduced by 40%, resulting in hypoxia of the nerve end membrane and accumulation of lactic acid
Indirect effect: Immune regulation	Low temperature inhibits surface CD80/CD86 of dendritic cells → initial T cell activation is blocked; activates NLRP3 inflammasome → macrophages release IL-1β/IL-18	DC, NLRP3, IL-1β, IL-18	This aggravates the local inflammatory response, leading to the formation of “inhibitory immunity + chronic low-grade inflammation”
Age-specific amplification effect	Initial T cells decrease by 60%-70% → excessive activation of memory T cells → increased secretion of IL-4/IL-13; NLRP3 of macrophages remains continuously activated	Initial T cells, memory T cells, IL-4, IL-13, NLRP3	The basal levels of pro-inflammatory factors (IL-6, TNF-α) increase by 2–3 times

TRPM8, Temperature sensitive Transient Receptor Potential Melastatin 8; DC, Dendritic Cells; NLRP3, NOD-like Receptor Protein 3; CD80, Cluster of Differentiation 80; CD86, Cluster of Differentiation 86; IL-1β, Interleukin-1 beta; IL-18, Interleukin-18; IL-4, Interleukin-4; IL-13, Interleukin-13; TNF-α, Tumor Necrosis Factor-α.

### 3.1 Definition and function of Th1/Th2 cells

Th1 and Th2 cells are two key subsets of CD4+ T cells, with distinct differentiation directions and functions (18). Th1 cells are characterized by the secretion of interferon-γ (IFN-γ) and interleukin-2 (IL-2), mainly mediating cellular immunity, and exerting functions such as anti-viral, anti-tumor, and clearance of intracellular pathogens by activating macrophages and cytotoxic T cells (19). Th2 cells, on the other hand, secrete IL-4, IL-5, and IL-13, dominating humoral immunity, promoting B cell proliferation and differentiation into plasma cells and the production of IgE antibodies, and recruiting eosinophils to participate in anti-parasitic infection and allergic reactions. Under physiological conditions, Th1/Th2 maintain immune homeostasis through cross-inhibition, for example, IFN-γ inhibits Th2 differentiation, while IL-4 inhibits Th1 function (20).

### 3.2 Effects of low temperature on Th cell differentiation: biased regulation of signal pathways

Low temperature (such as exposure at 4 °C) directly disrupts the Th1/Th2 balance by interfering with key transcription factor signaling pathways (21). Th1 differentiation depends on STAT1 phosphorylation, while low temperature can inhibit the activity of the JAK-STAT1 pathway, resulting in reduced IFN-γ secretion; at the same time, low temperature activates the STAT6 pathway (downstream signal of the IL-4 receptor), promotes the expression of GATA3 transcription factor, and enhances Th2 cell differentiation (22). Animal experiments show that in the spleen cells of mice exposed to low temperature, the expression of IL-4 is 2.3 times higher than that of the normal temperature group, while IFN-γ decreases by 40%, indicating Th2 polarization. In addition, low temperature can also upregulate the expression of the co-stimulatory molecule OX40L on the surface of antigen-presenting cells, further amplifying Th2-type immune responses (23).

### 3.3 Th1/Th2 imbalance in the elderly population: synergistic effect of immune aging and chronic inflammation

The core feature of immune aging in the elderly is a more significant decline in Th1 function. Thymus atrophy leads to a reduction in the initial T cell pool, and the proportion of memory T cells among the remaining T cells increases (>70%), while their ability to secrete IFN-γ decreases by 50%-60% compared to the younger population (24). At the same time, elderly individuals often have chronic low-grade inflammation (such as atherosclerosis, type 2 diabetes), and their macrophages and dendritic cells continuously secrete pro-inflammatory factors such as IL-6 and TNF-α, which indirectly promote Th2 differentiation by activating STAT6 and GATA3 (25). Clinical data show that the serum IL-4 level of elderly patients with facial paralysis is 1.8 times higher than that of healthy peers of the same age, while IFN-γ decreases by 30%, and the IL-4/IFN-γ ratio is significantly higher than that of young patients (P<0.01) (26). This “relative Th2 dominance” state makes the elderly population more prone to excessive inflammatory responses around the facial nerve under low-temperature stimulation, ultimately leading to demyelination and axonal damage (**Table 2**; **Figure 1**).

**Table 2:** The mechanism of Th1/Th2 imbalance and facial nerve inflammation association.

Imbalance type	Changes in cytokines	Immune cell behavior	Pathological manifestations of the facial nerve
Th2 dominant pro-inflammatory effect	IL-4, IL-13 ↑; IL-5↑	Activation of microglia → Release of ROS/NO; Infiltration of eosinophils → Release of MBP	Axonal lipid peroxidation, demyelination (demyelination thickness reduced by 30%)
Th1 inhibitory negative impact	IFN-γ↓; IL-2↓	Decreased ability of macrophages to clear pathogens; Reduction of Treg cell count by 25%	Increased risk of viral/bacterial colonization; CD4+ T cells continuously attack the nerve
Age-specific pathological changes	The IL-4/IFN-γ ratio increased by 1.8 times; the TNF-α level also increased by 1.8 times.	Overactivation of memory T cells → Inflammatory factor storm; Increase in apoptosis of Schwann cells	Extraneural membrane edema, compression of blood vessels (blood flow reduced by 50%), conduction block

IL-4, Interleukin-4; IL-13, Interleukin-13; IL-5, Interleukin-5; ROS, Reactive Oxygen Species; NO, Nitric Oxide; MBP, Major Basic Protein; IFN-γ, Interferon-γ; IL-2, Interleukin-2; Treg cell, Regulatory T Cell; CD4, Cluster of Differentiation 4; TNF-α, Tumor Necrosis Factor-α.

**Figure 1:**
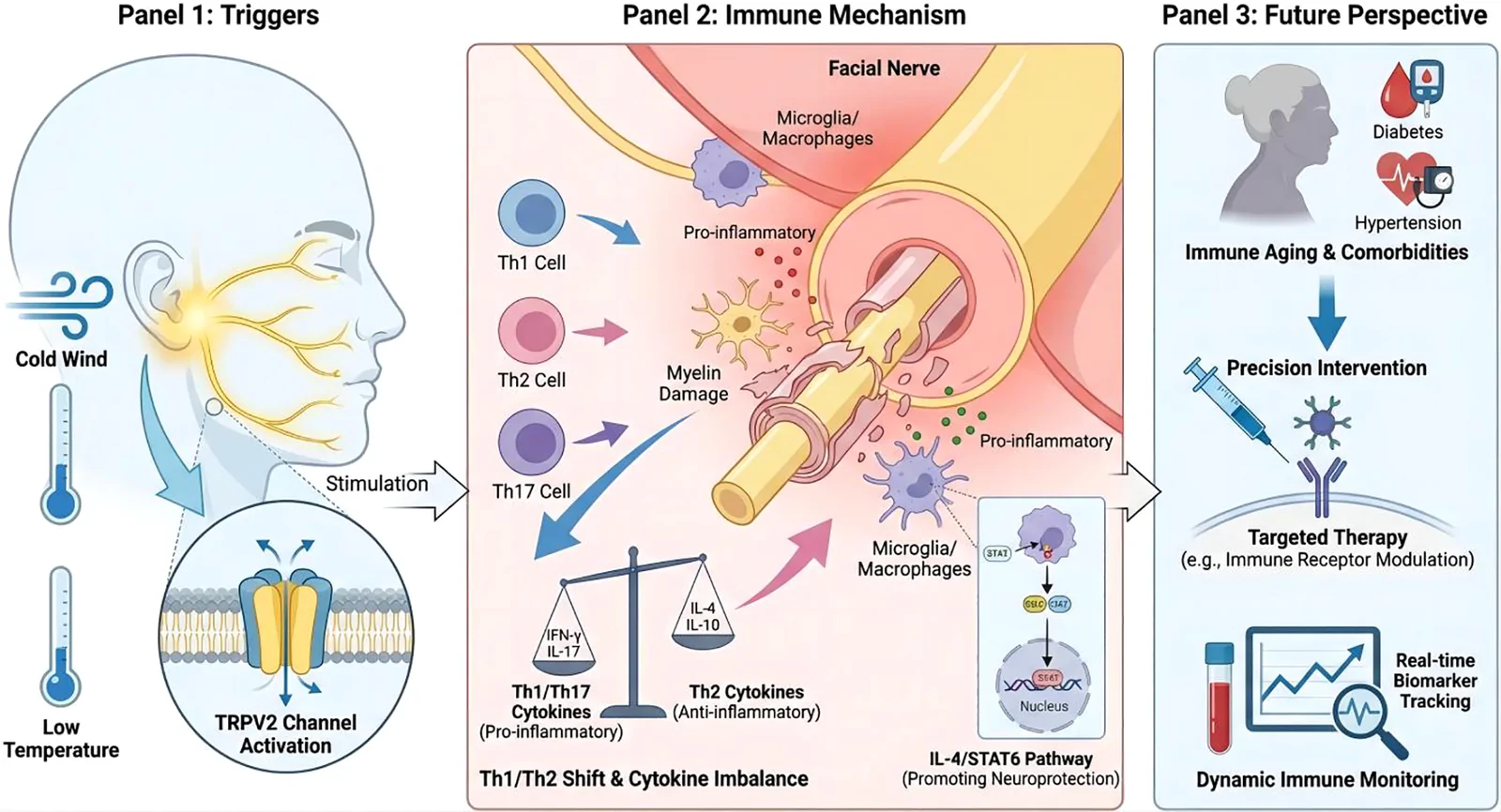
The mechanism of immune activation induced by low temperature in the pathogenesis of elderly IFP.

## 4 The association between Th1/Th2 imbalance and facial nerve inflammation

### 4.1 Pro-inflammatory effect of Th2 dominance: direct attack on the nerve

Th2 polarization induced by low temperature directly drives the inflammatory response around the facial nerve through the release of characteristic cytokines (IL-4, IL-5, IL-13) (27). IL-4 and IL-13 bind to the IL-4Rα receptor on the surface of microglia, activating the JAK-STAT6 pathway, causing microglia to transform into a pro-inflammatory phenotype and releasing a large amount of reactive oxygen species and nitric oxide. These oxidative stress substances can penetrate the neuronal membrane, causing axonal lipid peroxidation, protein nitration, and resulting in impaired nerve conduction function (28). IL-5 recruits eosinophils to the vicinity of the facial nerve through chemotaxis, and its main basic protein has strong toxicity, which can directly destroy the myelin structure and cause demyelination changes. Animal experiments show that in the facial nerves of Th2-polarized mice, the myelin thickness is reduced by 30% compared to the control group, and the nerve conduction velocity decreases by 45% (29).

### 4.2 Negative impact of Th1 inhibition: dual deficiency of immune defense and regulation

The decline of Th1 function leads to a decrease in IFN-γ secretion, weakening the pathogen clearance ability of macrophages (30). If there is a potential infection (such as a latent herpes simplex virus) around the facial nerve, the low-level IFN-γ cannot effectively activate the phagocytic function of macrophages, leading to viral replication or bacterial colonization, and exacerbating nerve damage. At the same time, the lack of IL-2 hinders the expansion of Treg, disrupting autoimmune tolerance (31). Treg can inhibit the excessive activation of effector T cells by secreting IL-10 and TGF-β, and a decrease in their number will cause CD4+ T cells to continuously attack the facial nerve, forming a vicious cycle of “immune attack - tissue damage - more immune infiltration”.

### 4.3 Pathological manifestations of facial nerve inflammation: dual collapse of structure and function

The imbalance of Th1/Th2 eventually leads to typical inflammatory pathological changes in the facial nerve: Infiltration of inflammatory cells around blood vessels, forming a “sock-like” structure; Excessive edema of the nerve’s outer membrane compresses the blood vessels, reducing local blood flow by more than 50%, causing ischemic damage; Apoptosis of Schwann cells (activated through the Caspase-3 pathway) leads to disruption of myelin regeneration, and nerve conduction blockage (32). Clinical biopsy shows that in elderly patients with facial paralysis, the number of apoptotic Schwann cells in the facial nerve is 2.1 times that of young patients, and the accumulation of myelin fragments is significant (33). These pathological changes jointly result in denervation of facial muscles, with clinical manifestations such as disappearance of forehead wrinkles and deviation of the mouth corner.

## 5 Specificity of the elderly population

### 5.1 Exhaustion of the T cell pool: a dual imbalance between antigen response capacity and inflammatory risk

The core feature of immune aging in the elderly is the progressive depletion of the initial T cell pool. Thymic atrophy leads to a 3%-5% reduction in the annual output of initial T cells, and in individuals over 60 years old, the proportion of initial T cells in peripheral blood is less than 20% (34). This reduction directly weakens the ability to recognize and respond to new antigens (such as those induced by low temperature in the facial nerve), making elderly individuals more prone to immune tolerance breakdown after exposure to low temperatures (35). At the same time, the proportion of memory T cells significantly increases, and they are mainly of the terminally differentiated effector memory T cells. Although these cells can rapidly release inflammatory factors (such as IFN-γ, TNF-α), their antigen specificity is narrow, and they are prone to over-activation under continuous low-temperature stimulation, leading to a “memory inflammatory factor storm” (36). Clinical data show that the serum TNF-α level in elderly patients with facial paralysis is 1.8 times higher than that in young patients, and it is positively correlated with the degree of facial nerve injury.

### 5.2 Disruption of the cytokine network: imbalance between pro-inflammatory and anti-inflammatory responses

The cytokine network in the elderly population shows a “pro-inflammatory - anti-inflammatory” imbalance (37). On one hand, aged macrophages and monocytes continuously secrete pro-inflammatory factors such as IL-6 and TNF-α, with their basal levels being 2–3 times higher than those in younger individuals. On the other hand, the secretion capacity of regulatory cytokines (such as IL-10) decreases, resulting in an anti-inflammatory/pro-inflammatory ratio (IL-10/TNF-α) dropping from 0.8 in younger individuals to below 0.3 (38). Cold exposure further exacerbates this imbalance: Cold exposure activates the NLRP3 inflammasome, increasing IL-1β secretion by 40%, while simultaneously inhibiting STAT3 phosphorylation, leading to a 25% reduction in IL-10 production. This state of “continuous release of pro-inflammatory factors + insufficient anti-inflammatory factors” makes the peripheral facial inflammation response difficult to control, resulting in chronic damage.

### 5.3 The cumulative effect of comorbidities: synergistic effects of multiple system impairments

The elderly population often suffers from multiple chronic diseases, which further amplifies the immunosuppressive effect of low temperatures (**Table 3**). The hyperglycemic state of diabetic patients can inhibit the function of Treg cells, reducing their ability to suppress effector T cells by 30%, while promoting the secretion of autoantibodies by B cells, thereby exacerbating the autoimmune attack on the facial nerve (39). The uremic toxins of patients with chronic kidney disease can activate dendritic cells and promote the differentiation of Th17 cells. The IL-17 secreted by them, in conjunction with Th2-type cytokines (IL-4, IL-13), can lead to a mixed inflammatory response of “Th2 + Th17” around the facial nerve (40). Animal experiments have shown that in elderly mice with diabetes exposed to low temperatures, the degree of demyelination of the facial nerve is 50% more severe than that in the simple low-temperature group, suggesting that comorbidities accumulate immune damage through multiple pathways (41). The immunosenescence of the elderly population and their comorbidity status make them a high-risk group for low-temperature-related facial nerve damage. For this group, more proactive preventive measures are needed, such as avoiding prolonged exposure to low temperatures, controlling blood sugar and renal function, and exploring intervention strategies targeting cytokine networks or regulating T cell balance.

**Table 3:** The synergistic effect of immune aging and comorbidities in the elderly population on cold injury.

Factor classification	Immune aging characteristics	Comorbidity effects	The combined effects after exposure to low temperature
T cell depletion	The proportion of naive T cells is less than 20%; the proportion of memory T cells is more than 70% (predominantly terminal differentiation type)	Diabetes: Hyperglycemia inhibits Treg function → Increase in secretion of autoantibodies (anti-GQ1b)	The risk of immune tolerance breakdown increases; antigen-specificity narrows → “memory inflammatory storm”
Disorder of the cytokine network	The basal levels of pro-inflammatory factors (IL-6, TNF-α) increase by 2–3 times; the ratio of anti-inflammatory/pro-inflammatory (IL-10/TNF-α) drops below 0.3	Chronic kidney disease: Uremic toxins activate DC → Increase in Th17 cell differentiation (IL-17 secretion)	Continuous release of pro-inflammatory factors + insufficient anti-inflammatory response → chronic facial nerve damage
Comorbidity superimposition effect	The decline of immune system function coexists with chronic inflammation	Diabetes + Chronic kidney disease: Mixed Th2 + Th17 inflammatory response	The degree of demyelination of the facial nerve worsens by 50%; synergistic amplification effect of multiple system injuries

Treg, Regulatory T Cell; anti-GQ1b, Anti-Ganglioside Q1b Antibody; IL-6, Interleukin-6; TNF-α, Tumor Necrosis Factor-α; IL-10, Interleukin-10; DC, Dendritic Cell; Th17, T Helper 17 Cell; IL-17, Interleukin-17.

Treg, Regulatory T Cell; anti-GQ1b, Anti-Ganglioside Q1b Antibody; IL-6, Interleukin-6; TNF-α, Tumor Necrosis Factor-α; IL-10, Interleukin-10; DC, Dendritic Cell; Th17, T Helper 17 Cell; IL-17, Interleukin-17.6 Evaluation of comorbidity factors and therapeutic interactions.

Patients with IFP often have multiple chronic diseases, such as diabetes, chronic kidney disease, autoimmune diseases, etc., and may also receive treatments such as corticosteroids, antiviral drugs, etc. These comorbidity factors and therapeutic approaches may significantly alter the risk of IFP occurrence, the degree of inflammation, and prognosis (42). For instance, diabetes can exacerbate the immune imbalance induced by low temperature. The hyperglycemic state inhibits the function of Treg, reducing their inhibitory effect on effector T cells, thereby intensifying the excessive secretion of Th2-type cytokines (such as IL-4, IL-13) (43). Among patients with chronic kidney disease, the accumulated uremic toxins in the body can activate dendritic cells, promoting the differentiation of Th17 cells, resulting in increased IL-17 secretion. IL-17 and Th2-type cytokines (IL-4, IL-13) work synergistically to form a “Th2 + Th17” mixed inflammatory response, exacerbating the damage to the facial nerve surrounding tissues. Long-term use of corticosteroids (such as prednisone) can inhibit the activation of initial T cells and reduce IFN-γ secretion, causing a shift towards a Th2-dominated balance. Although corticosteroids can alleviate the acute phase of facial nerve edema, long-term use reduces the ability to clear pathogens and increases the risk of latent viruses (such as herpes simplex virus) reactivation (44). Moreover, corticosteroids can also induce hyperglycemia, forming a vicious cycle with diabetes, further exacerbating immune imbalance. Future studies should conduct prospective cohort studies to clarify the impact of comorbidity factors on the long-term prognosis of IFP. By integrating syndrome management, immune monitoring and individualized treatment, it is expected to significantly improve the clinical prognosis of elderly patients with IFP and reduce the risk of recurrence.

## 7 Discussion

This review focuses on the association between low temperature and facial nerve inflammation, and discovers that low temperature inhibits the differentiation of Th1 cells, reduces the secretion of IFN-γ, and promotes the polarization of Th2 cells, increases the release of IL-4 and IL-13, thereby disrupting the Th1/Th2 immune balance and triggering excessive inflammation around the facial nerve (45). In the elderly population, due to immune aging, the initial T cell count declines and the function of memory T cells becomes abnormal, along with common comorbidities such as diabetes and chronic kidney disease, they are more sensitive to low temperature stimulation (46). The Th2 dominance is more prominent, the ratio of IL-4 to IFN-γ increases, and facial nerve damage is more severe, with phenomena such as demyelination and axonal degeneration occurring (47). This finding has established a theoretical framework for analyzing the immunological pathogenesis of facial paralysis in the elderly, highlighting the crucial role of age-related immune changes in the disease progression.

In the discussion of the relationship between low temperature and facial paralysis, there has always been a controversy over whether low temperature directly damages the facial nerve. After integrating multiple clinical and basic research results, this review clearly states that the immune-mediated inflammatory response is the dominant mechanism by which low temperature induces facial paralysis (48). It is found that in patients exposed to low temperature, the biopsy results of the facial nerve show a large number of CD4+ T cells and eosinophils infiltrating; in contrast, the isolated nerve specimens treated only with low temperature did not show obvious axonal degeneration (49). This result strongly supports the hypothesis that low temperature causes immune imbalance and indirectly damages the nerve.

In the research on IFP, the impact of comorbidities on the prognosis of the disease cannot be ignored. Diabetes and chronic kidney disease are like two “boosters”, amplifying the immune effects caused by low temperatures through multiple pathways, making the damage to the facial nerve even more severe (50). The long-term high blood sugar state in diabetic patients inhibits Treg cells with immune regulatory functions, causing excessive activation of Th2 cells and triggering abnormal immune responses; while in patients with chronic kidney disease, uremic toxins such as indoxyl sulfate induce the differentiation of Th17 cells, which, in conjunction with Th2 cells, further exacerbates inflammation (51). It is precisely these interactions between the comorbidities that lead to poor prognosis for elderly patients with facial paralysis, providing important evidence for clinicians to intervene from the perspective of comorbidity management.

In the clinical translation and future exploration of elderly facial paralysis, there are new directions in diagnosis, treatment, and prevention. In terms of diagnosis, the serum IL-4/IFN-γ ratio can serve as a prognostic biomarker, which can guide treatment and predict recovery (52). In terms of treatment, intervention strategies targeting the Th2 pathway, such as anti-IL-4 antibodies, are expected to become new therapies, but their applicability in facial nerve inflammation needs to be explored (53). Given the sensitivity of the elderly population to low temperatures and its close association with the occurrence of IFP, we emphasize the importance of implementing personalized cold protection strategies. In terms of indoor environment, it is recommended to maintain the indoor temperature at 22-24°C in winter, and use local heating equipment to avoid direct cold wind stimulation on the face (54). For the selection of warm clothing, it is recommended to wear a warm mask or scarf to cover the ear and jaw areas, and prefer materials that are breathable and windproof. In terms of outdoor management, during low-temperature weather (<10°C), the duration of outdoor activities should be limited, especially avoiding activities during the low-temperature periods in the early morning or evening (55). Before going out, facial heat application (5 minutes, 40°C) can be performed to enhance local cold resistance and thereby reduce direct damage to the facial nerve from low temperatures. Additionally, the clinical application of interventional immunomodulatory measures also requires further exploration. For patients with severe Th2 polarization, short-term use of anti-IL-4 monoclonal antibodies may be an effective treatment method, but infection risks need to be closely monitored. Additionally, Treg cell expansion therapy is suitable for patients with a significantly elevated IL-4/IFN-γ ratio and chronic inflammation (such as diabetes), by infusing autologous Treg cells to inhibit excessive Th2 responses, thereby reducing nerve inflammation. The timing of the use of these immunomodulatory measures is crucial, and it is recommended to start within 72 hours after the onset of the disease to block the inflammatory cascade and prevent further deterioration of nerve damage (45). Furthermore, for the management of comorbidities, we emphasize the importance of multidisciplinary collaboration. For patients with diabetes, strict control of blood glucose levels (HbA1c < 7%) is necessary to reduce Treg function inhibition and the risk of facial nerve damage. For patients with chronic kidney disease, regular monitoring of uremic toxin levels is required. If the levels are elevated, intensified hemodialysis treatment is necessary to reduce the mixed inflammatory response induced by Th17 cell differentiation, thereby protecting the facial nerve from further damage (56). In conclusion, future prospective cohort studies should be conducted to dynamically monitor immune indicators and explore the interaction mechanism between immune aging and comorbidities, providing a basis for precise intervention.
